# Systematic review and survey of *Neisseria gonorrhoeae* ceftriaxone and azithromycin susceptibility data in the Asia Pacific, 2011 to 2016

**DOI:** 10.1371/journal.pone.0213312

**Published:** 2019-04-03

**Authors:** C. R. Robert George, Rodney P. Enriquez, Barrie J. Gatus, David M. Whiley, Ying-Ru Lo, Naoko Ishikawa, Teodora Wi, Monica M. Lahra

**Affiliations:** 1 WHO Collaborating Centre for Sexually Transmitted Infections and Antimicrobial Resistance, New South Wales Health Pathology, Microbiology, Prince of Wales Hospital, Randwick, New South Wales, Australia; 2 New South Wales Health Pathology, Microbiology, Prince of Wales Hospital, Randwick, New South Wales, Australia; 3 Faculty of Medicine, UQ Centre for Clinical Research, The University of Queensland, Herston, Queensland, Australia; 4 Pathology Queensland, Microbiology Department, Herston, Queensland, Australia; 5 World Health Organization, Office for Malaysia, Brunei Darussalam and Singapore, Kuala Lumpur, Malaysia; 6 World Health Organization, Regional Office for the Western Pacific, Manila, Philippines; 7 Department of Reproductive Health and Research, World Health Organization, Geneva Switzerland; 8 School of Medical Sciences, The University of New South Wales, Sydney, New South Wales, Australia; Emory University School of Medicine, UNITED STATES

## Abstract

**Background:**

Antimicrobial resistance in *Neisseria gonorrhoeae* is a global concern, with the ongoing emergence of ceftriaxone and azithromycin resistance threatening current treatment paradigms. To monitor the emergence of antimicrobial resistance in *N*. *gonorrhoeae*, the World Health Organization (WHO) Gonococcal Antimicrobial Surveillance Programme (GASP) has operated in the Western Pacific and South East Asian regions since 1992. The true burden of antimicrobial resistance remains unknown. In response, the objective of this study was to survey ceftriaxone and azithromycin susceptibility in *N*. *gonorrhoeae* across the western Pacific and south-east Asia, and interlink this data with systematically reviewed reports of ceftriaxone and azithromycin resistance.

**Methods and findings:**

The WHO Collaborating Centre for Sexually Transmitted Infections and Antimicrobial Resistance, Sydney, coordinated annual surveys of gonococcal susceptibilities with participating laboratories, and additionally undertook a systematic review of reports detailing gonococcal ceftriaxone and azithromycin susceptibility data for locations geographically in the Asia Pacific from 2011 to 2016. It was found that surveillance of gonococcal antimicrobial resistance remains limited in the Asia Pacific, with weaker surveillance of azithromycin versus ceftriaxone. Ninety-three published reports were identified (including national reports) which documented susceptibility data for ceftriaxone and azithromycin. GASP survey data was available for 21 countries, territories or areas, and suggested MICs are increasing for ceftriaxone and azithromycin. Between 2011 and 2016, the percentage of locations reporting >5% of gonococcal isolates with MICs to ceftriaxone meeting WHO’s definition of decreased susceptibility (MIC ≥ 0.125 mg/L) increased from 14.3% to 35.3% and the percentage of locations reporting >5% of gonococcal isolates with azithromycin resistance (MIC ≥ 1 mg/L) increased from 14.3% to 38.9%. Published reports were available for several countries that did not provide GASP surveillance responses for ceftriaxone (n = 5) and azithromycin (n = 3) respectively. Over the study period, there was a 183% increase in the number of countries providing surveillance data for GASP for both ceftriaxone and azithromycin, and a 30.6% increase in ceftriaxone MIC testing across the Asia Pacific facilitated by this project.

**Conclusion:**

This study provides the first comprehensive illustration of increasing MICs to ceftriaxone in the Asia Pacific. The survey and literature review additionally detail increasing resistance to azithromycin. Further surveillance system strengthening is required to monitor these trends in order to address and curb gonococcal AMR in the region.

## Introduction

The development of antimicrobial resistance in *Neisseria gonorrhoeae* is of global concern [1], with increased ceftriaxone non-susceptibility, and reports of ceftriaxone resistant strains in Japan, France, Spain, and Australia [2]. Over the past 100 years, *N*. *gonorrhoeae* has established resistance to every class of clinically tested antimicrobial agent [3]. Currently many countries recommend dual therapy treatment using the extended spectrum cephalosporin ceftriaxone and the macrolide azithromycin. However, as recognized in the WHO report *Antimicrobial resistance*: *global report on surveillance 2014*, there are significant gaps in *N*. *gonorrhoeae* antimicrobial resistance surveillance, particularly where disease rates are highest [4]. Of concern, azithromycin resistance has been increasing since first being documented in the 1990s, and the past decade has seen the emergence of ceftriaxone resistant strains including H041 (Japan, 2009), F89 (France, 2010; Spain, 2011), A8806 (Australia, 2013), GU140106 (Japan, 2014), and FC428 and FC460 (Japan, 2015); however, the true extent of this emergence remaining largely unknown [1, 4–6]. The quality of antimicrobial surveillance in the Asia Pacific is of particular interest given apparently higher overall rates of resistance compared with North America and Europe; of concern, however, is rate at which global transportation of such strains permits their spread globally. Numerous factors influence antimicrobial surveillance efforts, including resources, and political and technical constraints (e.g., sample viability during transport, availability of skilled technicians able to perform susceptibility testing). Interplay between such factors at local, national, regional and international levels contributes to the gaps and deficits in AMR testing, surveillance and reporting.

Globally, there is great variability between the quality and intensity of antimicrobial surveillance for WHO priority organisms such as *N*. *gonorrhoeae* [4]. For optimal surveillance of antimicrobial resistance in *N*. *gonorrhoeae*, minimum inhibitory concentrations (MICs) are required. Agar dilution and gradient diffusion methodologies (such as the bioMérieux Etest) are available, with the latter being methodologically straightforward whilst providing indicative MIC values. Disc diffusion methodologies, whilst straightforward and accessible in resource limited settings, cannot provide MIC values. There is a consequent need to establish sustainable methods for MIC testing particularly in resource limited settings.

Assessment of the WHO Gonococcal Antimicrobial Surveillance Programme (GASP) laboratories in 2012 indicated that many regional laboratories were limited to disc testing where MIC data were required in particular for ceftriaxone, given disc data poorly captures incremental changes of MICs. In response, the WHO Collaborating Centre for Sexually Transmitted Infections and Antimicrobial Resistance (WHO CC), Sydney, implemented the NG MIC Snapshot Study to expand GASP to include ceftriaxone MIC data, to inform public health strategies, and to enhance regional surveillance and identify surveillance gaps.

This study’s goal was to perform a situation analysis and to collect MIC data for the GASP. The study integrated with the GASP’s pre-existing regional surveillance systems to pilot strip diffusion MIC testing in laboratories unable to perform agar dilution methods. This provided quality assured data describing resistance rates for *N*. *gonorrhoeae* at minimal additional burden to regional resources. To maximise detection of reported ceftriaxone and azithromycin susceptibility data, a multimodal approach building on the WHO’s *Antimicrobial resistance*: *global report on surveillance 2014* was adopted whereby we examined GASP data and also reviewed national reports and published literature for the Western Pacific Region (WPR) and South-East Asian Region (SEAR) [4].

This report describes the literature review and surveys conducted by the WHO CC, Sydney, which is the Regional GASP coordinating laboratory for the WPR, over the period 2011 through 2016, which corresponds to the most recent complete survey data presently available. The objective was to identify all reports detailing ceftriaxone and azithromycin susceptibility from the Asian Pacific region, and to interlink this with the GASP data expanded by using strip diffusion MIC testing to identify gaps in surveillance. The study was intended to complement the objectives of the Global Antimicrobial Resistance Surveillance System (GLASS).

## Methods

### Systematic review

A systematic review was performed to identify reports detailing ceftriaxone and azithromycin susceptibility data for *N*. *gonorrhoeae* in the study regions. A standardized protocol was followed. We searched PubMed, Embase, and Web of Science databases for articles published between 1 January 2011 and 31 December 2016. No language restrictions were used, and articles written in non-English languages were translated to English before review. Search terms included the name of each location geographically within the WPR and SEAR, and at least one of “gonococcus” or “gonorrhea” or “gonorrhoea” or “gonorrhoeae”. Locations (and their associated search terms) geographically within the SEAR and WPR included WPR members: Australia (“Australia”), Brunei (“Brunei”), Cambodia (“Cambodia”), China (“China”), Cook Islands (“Cook Islands”), Fiji (“Fiji”), Japan (“Japan”), Kiribati (“Kiribati”), South Korea (“South Korea”), Lao (“Lao”), Malaysia (“Malaysia”), Marshall Islands (“Marshall Islands”), Micronesia (“Micronesia”), Mongolia (“Mongolia”), Nauru (“Nauru”), New Zealand (“New Zealand”), Niue (“Niue”), Palau (“Palau”), Papua New Guinea (“Papua New Guinea”), Philippines (“Philippines”), Samoa (“Samoa”), Singapore (“Singapore”), Solomon Islands (“Solomon Islands”), Tonga (“Tonga”), Tuvalu (“Tuvalu”), Vanuatu (“Vanuatu”), Viet Nam (“Vietnam”); SEAR members: Bangladesh (“Bangladesh”), Bhutan (“Bhutan”), India (“India”), Indonesia (“Indonesia”), North Korea (“North Korea”), Maldives (“Maldives”), Myanmar (“Myanmar”), Nepal (“Nepal”), Sri Lanka (“Sri Lanka”), Thailand (“Thailand”), Timor-Leste (“East Timor”); and other sites including locations reporting to other entities: Christmas Island (Australia; “Christmas Island”), Cocos Keeling Islands (Australia, “Cocos Keeling Islands”), Norfolk Island (Australia, “Norfolk Island”), Taiwan (“Taiwan”), American Samoa (United States, “American Samoa”), French Polynesia (France, “French Polynesia”), Guam (United States, “Guam”), Hong Kong (China, “Hong Kong”), Macao (China, “Macau”), New Caledonia (France, “New Caledonia”), Northern Mariana Islands (United States, “Northern Mariana Islands”), Pitcairn Islands (United Kingdom, “Pitcairn Islands”), Tokelau (New Zealand, “Tokelau”), and Wallis and Futuna (France, “Wallis and Futuna”). Where possible, search locations were mapped (e.g., as a MeSH term). A supplemental grey literature search was performed using http://www.google.com.au to identify additional surveillance reports from locations within the SEAR and WPR not detected using the indexed databases. Search terms were structured using the format “intitle:"<country name>" ("gonococcus" OR "gonorrhea" OR "gonorrhoea" OR "gonorrhoeae")” and all returned results were reviewed for relevance.

Data were extracted from reports by an investigator (CRRG) using duplicate review strictly in accordance with inclusion and exclusion criteria. Reports were included if they contained ceftriaxone or azithromycin susceptibility data from one or more isolates of *N*. *gonorrhoeae* collected between 2011 and 2016. Qualitative and quantitative susceptibility data were interpreted in context of the testing methodology utilized (e.g., Clinical & Laboratory Standards Institute [CLSI], European Committee on Antimicrobial Susceptibility Testing [EUCAST], Calibrated Dichotomous Sensitivity [CDS]; disc testing, MIC by gradient diffusion or agar dilution; and quantitative MIC data versus dichotomous susceptibility data). Studies were excluded if they: did not contain primary data collection or described susceptibility data identifiably published elsewhere (excluding cases where national reports potentially described isolates published elsewhere); did not specify the collection dates of analyzed samples; or if they did not explicitly state the country of isolate collection. Studies referencing infected travelers diagnosed outside the study area were excluded. The variables assessed were: location, date of testing, the testing method, and antimicrobial susceptibility testing result. All publication types were potentially eligible for inclusion.

Reports were initially screened for relevance, and all potentially relevant reports analyzed to determine if they satisfied the aforementioned inclusion versus exclusion criteria. No specific analysis was performed to assess for reporting bias within publications, given any report of susceptibility data was considered relevant and mapping ranges were not established from published data.

For the purposes of this review, a ‘national report’ was defined as a document containing national level surveillance data and derived from an agency that was recognisably designated by its country as the national authority for the purposes of reporting ongoing *N*. *gonorrhoeae* surveillance data. All other reports not meeting the criteria of a national report were classified as ‘publications.’

### GASP survey data

In 1992, the WHO CC, Sydney established an antimicrobial susceptibility testing surveillance programme for *N*. *gonorrhoeae* for the WPR and SEAR, which it has coordinated since that time. The survey initially focussed on ciprofloxacin and penicillin, with subsequent surveillance of ceftriaxone and azithromycin. The WHO CC, Sydney supports regional laboratories through provision of technical advice and support, some supplies, an external quality assurance programme, training, and laboratory training on request. Annually, GASP participants submit quality assured annual surveillance data to the WHO CC, Sydney for the WHO, which is collated based on site of collection and submitted to the WHO for inclusion in the GASP. In Australia, because of substantial differences in resistance profiles in remote populations compared with non-remote populations, data is de-aggregated and reported separately. For all other sites, survey data is collated at the country or territory level (herein referred to as ‘country level reports’).

The present study collated survey results for ceftriaxone and azithromycin (ciprofloxacin and penicillin are included for reference purposes). The percentage of isolates meeting the WHO’s definition of decreased susceptibility to ceftriaxone (MIC ≥ 0.125 mg/L) and/or resistance to azithromycin (MIC ≥ 1.0 mg/L) were calculated as a function of the total number of isolates tested at each site, and mapping ranges assigned to this data based on previously used WHO criteria [7]. The study utilised MIC data for ceftriaxone derived from supplied Etest strips (bioMérieux, Marcy I'Etoile, France).

### Synthesis

EUCAST, CLSI and CDS variously define azithromycin and ceftriaxone susceptibility, resulting in variable terminology use within the published literature. Regarding azithromycin susceptibility, EUCAST defines ‘susceptible’ (MIC ≤ 0.25 mg/L), ‘intermediate’ (MIC = 0.5 mg/L) and ‘resistant’ (MIC > 0.5 mg/L) [8]. CDS defines azithromycin as ‘sensitive’ (MIC < 1.0 mg/L), or ‘resistant’ (MIC ≥ 1.0 mg/L) [9]. Prior to 2017, CLSI did not provide guidance on azithromycin susceptibility, and has subsequently referred to Epidemiological Cutoff Values (ECVs), advising that these are not to be reported as susceptible, intermediate or resistant [10]. The North American Gonococcal Isolate Surveillance Project (GISP) defines azithromycin ‘reduced susceptibility’ (MIC ≥ 2.0 mg/L) [11]. Regarding ceftriaxone susceptibility, CLSI defines ‘susceptible’ (MIC ≤ 0.25 mg/L) and ‘nonsusceptible’ (any other MIC value) [10]. EUCAST categorises ceftriaxone MICs as being ‘susceptible’ (MIC ≤ 0.125 mg/L) and ‘resistant’ (MIC > 0.125 mg/L) [8]; CDS categorises ceftriaxone susceptibility as ‘sensitive’ (MIC < 0.06 mg/L) or ‘decreased susceptibility’ (MIC 0.06 to 0.25 mg/L), but does not define criteria for ‘resistant’ [9]. The WHO Global Action Plan 2012 defines ceftriaxone ‘decreased susceptibility’ (MIC ≥ 0.125 mg/L) [12]. For ceftriaxone, GASP reports utilise the MICs provided by each participant to determine how isolates would be categorised using WHO criteria of ‘decreased susceptibility’. Given the variety of definitions describing ceftriaxone and azithromycin susceptibility, for the purposes of this report, isolates were categorised as resistant to azithromycin if they met any of the following: EUCAST ‘resistant’, CDS ‘resistant’, or if an isolate was reported as being anything other than ‘sensitive’ or ‘susceptible’ (e.g., low level resistant). For ceftriaxone, given complexities in testing and interpretation, for the purposes of this report, isolates were considered to have an ‘alert level MIC’ if their MIC (or a surrogate measure of the MIC) was described as meeting the WHO criteria for decreased susceptibility (i.e., MIC ≥ 0.125 mg/L). The term ‘alert level MIC’ has been used given some isolates could potentially have MICs that greatly exceed 0.125 mg/L, and to avoid confusion with method-specific terminology used by EUCAST, CLSI, and CDS.

Locations geographically within the WPR and SEAR were categorised by the level of susceptibility reported, the type of testing performed (where identified), participation in the GASP network, and the types of published evidence obtainable, including national reports, or other literature accessible through indexed databases, or a through a grey literature search. Using this approach, the presence of isolates with alert level MICs for ceftriaxone or resistance to azithromycin were determined from the reviewed literature and GASP survey results for each location within the WPR and SEAR.

## Results

### Systematic review

**Fig 1:**
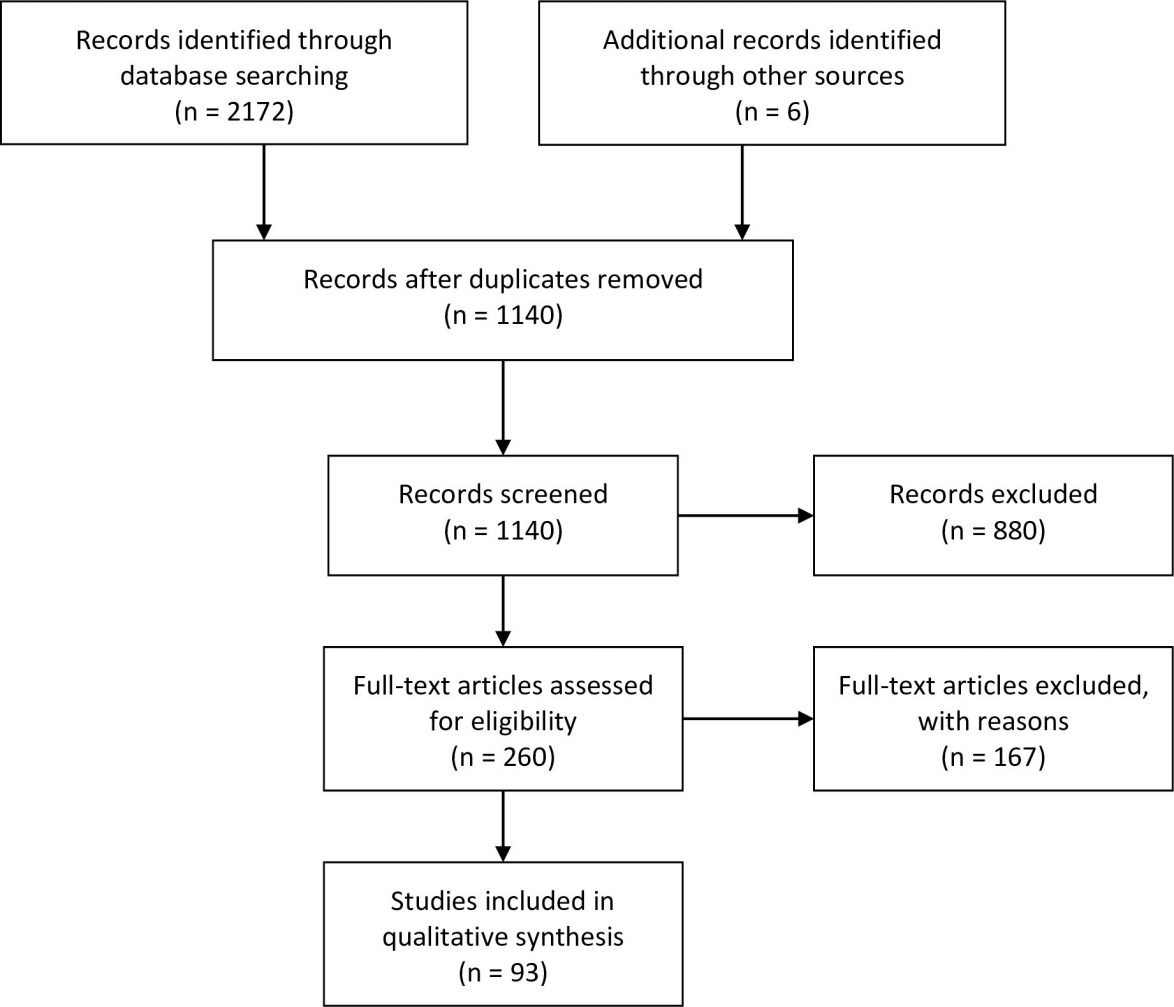
PRISMA flow diagram detailing inclusion and exclusion of records. 93 studies were included from 2172 records initially screened through database searching, and 6 records identified through supplemental searching.

Of 2172 records identified through database searching, and 6 additional records identified through supplemental search, 93 articles were included for qualitative analysis (Fig 1; Table 1). The most common reason for exclusion related to duplication (1591 removed; e.g., the same record returned from multiple databases, or same record returned for multiple countries), or due to irrelevance on screening (880 excluded).

**Table 1:** Reports detailing interpretations for ceftriaxone and azithromycin susceptibility testing between 2011 and 2016 in *Neisseria gonorrhoeae* from the South East Asian and Western Pacific regions, and geographically located sites for countries and territories not members of WPR or SEAR, or member states or territories that report to other WHO regional offices.

Region	Activities	Ceftriaxone	Azithromycin
South-East Asian	GASP responses	Bhutan, India, Myanmar, Sri Lanka, Thailand	Bhutan, India, Myanmar, Sri Lanka, Thailand
	National reports	Nil identified	Nil identified
	Other publications	Bangladesh [13–15], Bhutan [16], India [16–26], Indonesia [27, 28], Nepal [29], Sri Lanka [16], Thailand [16, 30, 31]	Bangladesh [14], Bhutan [16], India [16–21, 24, 25], Indonesia [27, 28], Nepal [29], Thailand [16, 30]
Western Pacific	GASP responses	Australia, Brunei, Cambodia, China, Japan, South Korea, Mongolia, New Caledonia, New Zealand, Philippines, Singapore, Viet Nam	Australia, Brunei, Cambodia, Japan, South Korea, Mongolia, New Zealand, Philippines, Singapore, Viet Nam
	National reports	Australia [32–45], New Zealand [46–48]	Australia [32–45]
	Other publications	Australia [49–62], Cambodia [63], China [64–79], Japan [80–94], Philippines [95], South Korea [96–98], Viet Nam [99]	Australia [49–51, 53, 54, 57–60, 62], China [69, 70, 72, 73], Japan [80–88, 91–94, 100, 101], South Korea [96, 98], Viet Nam [99]
Other	GASP responses	Hong Kong	Hong Kong
	National reports	Nil identified, although reporting could be incorporated into overseeing member state reports	Nil identified, although reporting could be incorporated into overseeing member state reports
	Other publications	Taiwan [102–105]	

WPR members: Australia, Brunei, Cambodia, China, Cook Islands, Fiji, Japan, Kiribati, South Korea, Lao, Malaysia, Marshall Islands, Micronesia, Mongolia, Nauru, New Zealand, Niue, Palau, Papua New Guinea, Philippines, Samoa, Singapore, Solomon Islands, Tonga, Tuvalu, Vanuatu, Viet Nam. SEAR members: Bangladesh, Bhutan, India, Indonesia, North Korea, Maldives, Myanmar, Nepal, Sri Lanka, Thailand, Timor-Leste. Others (including locations reporting to other sites): Christmas Island (Australia), Cocos Keeling Islands (Australia), Norfolk Island (Australia), Taiwan, American Samoa (United States), French Polynesia (France), Guam (United States), Hong Kong (China), Macao (China), New Caledonia (France), Northern Mariana Islands (United States), Pitcairn Islands (United Kingdom), Tokelau (New Zealand), Wallis and Futuna (France).

### Surveillance intensity

For the SEAR, five countries participated in the GASP between 2011 and 2016, with all participants reporting ceftriaxone and azithromycin survey results on at least one occasion (Table 2). Between 2011 and 2016, the number of isolates tested from SEAR annually ranged from 546 to 970 (average 762) for ceftriaxone, and 737 to 1144 (average 892) for azithromycin. A total of 4571 isolates were tested for ceftriaxone, and 5350 isolates tested for azithromycin (S1 Table). In 2016, all participants reported ceftriaxone and azithromycin susceptibility rates. Six countries did not participate in GASP. Of the 11 members of the SEAR, published data is available for the majority (ceftriaxone data is available for 7, azithromycin data is available for 6) (Table 1). The majority of published reports for the region related to Indian isolates. No national reports were identified.

**Table 2:** Rates of antimicrobial resistance obtained from *Neisseria gonorrhoeae* surveillance data submitted to the Gonococcal Antimicrobial Surveillance Programme.

	Ceftriaxone						Azithromycin						Ciprofloxacin						Penicillin					
	2011	2012	2013	2014	2015	2016	2011	2012	2013	2014	2015	2016	2011	2012	2013	2014	2015	2016	2011	2012	2013	2014	2015	2016
**WPR**																								
Australia (Non-remote)	1	1	1	1	1	1	1	1	1	1	1	1	**4**	**4**	**4**	**4**	**3**	**4**	**3**	**4**	**4**	**3**	**3**	**3**
Australia (Remote)	1	0	0	0	0	0	0	1	0	0	0	1	1	1	1	1	1	1	1	1	1	1	1	1
Brunei												1	**5**	**5**	**5**			**5**	**4**	**4**	**5**			**4**
Cambodia						*0*						***4***		***5***	***5***			***5***						***4***
China	**3**	**2**			**2**	**2**					**3**	**3**	**5**	**5**			**5**	**5**	**4**	**4**			**5**	**5**
Fiji													0	*0*	0				**2**	**2**	**3**			
Hong Kong [China]	1	**2**	1	1	1	1	1	1	**3**	1	**2**	**2**	**5**	**5**	**5**	**5**	**5**	**5**	**4**	**4**	**4**	**4**	**4**	**4**
Japan	1	1	**3**	**3**	**3**	**3**	**3**	**2**	**2**	**2**	**3**	**2**	**5**	**5**	**5**	**5**	**5**	**5**	**4**	**4**	**4**	**4**	**4**	**4**
Malaysia													**4**											
Mongolia						**3**			1	**2**	**2**	1		**4**	**3**	**4**	**4**	**5**			**4**	**4**	**4**	**5**
New Caledonia			0			0							**2**	1	1	1	**2**	**2**	**2**	**2**	**3**	1	1	1
New Zealand	0	0	1	1	1	1			0	1	1	**2**	**4**	**4**	**4**	**4**	**4**	**3**	**3**	**2**	**2**	**4**	**3**	**2**
Philippines		0	0	0	0	1			0	0	0	0	**5**	**5**	**5**	**5**	**5**	**5**	**5**	**5**	**5**	**5**	**5**	**5**
Singapore			1	0	0	**2**				1	1	1	**5**	**5**	**5**	**5**	**5**	**5**	**4**	**4**	**3**	**3**	**4**	**4**
South Korea			**2**	**2**	**3**	**2**		0	0	0	0	0	**5**	**5**	**5**	**5**	**5**	**5**	**4**	**4**	**4**	**4**	**5**	**4**
Viet Nam					**2**		1	1	1	1	0	0	**5**	**5**	**5**	**5**	**5**	**5**	**4**	**4**	**5**	**5**	**4**	**4**
**SEAR**																								
Bhutan				1	1	0		0	0	**2**	0	0	**5**	**5**	**5**	**5**	**5**	**5**	**5**	**5**	**5**	**5**	**5**	**5**
India			0	0	0	0	1	1	1	0	1	**2**	**5**	**5**	**5**	**5**	**5**	**5**	**4**	**5**	**4**	**4**	**4**	**4**
Myanmar						***4***						***3***	***5***					***5***	**4**					***5***
Sri Lanka				0		0			1	1	0	1	**5**	**5**	**5**	**5**	**5**	**5**	**5**	**5**	**5**	**5**	**5**	**4**
Thailand	0	1	1	1	0	0	1	1	1	1	1	1	**5**	**5**	**5**	**5**	**5**	**5**	**5**	**5**	**4**	**5**	**5**	**5**

Key: Percentage mapping range of isolates with: Alert level MICs for ceftriaxone (MIC ≥ 0.125), with data excluded for isolates tested using disc testing; Azithromycin resistant (MIC ≥ 1), ciprofloxacin resistant (MIC ≥ 1), and penicillin resistant (MIC ≥ 1). Rates of resistance (azithromycin, ciprofloxacin, penicillin) or alert level MICs (ceftriaxone): 0 = 0%, 1 = 0.1–5%, 2 = 6–15%, 3 = 16–30%, 4 = 31–70%, 5 = 71–100%. Italicised entries are based on ≤ 10 samples. Boldened entries exceed 5%.

For the WPR, GASP data was drawn from 15 countries and territories between 2011 and 2016, with Australian data being subcategorised as remote and non-remote. Ceftriaxone and/or azithromycin susceptibility data was available for 14 of these sites, and two countries provided data only on ciprofloxacin and/or penicillin (Table 2). Between 2011 and 2016, the number of isolates tested from WPR annually ranged from 6880 to 9763 (average 7942) for ceftriaxone, and 5630 to 9950 (average 7486) for azithromycin (S1 Table). A total of 47654 isolates were tested for ceftriaxone, and 44917 isolates tested for azithromycin. The majority of identified publications and national reports (87% providing ceftriaxone data, and 93% for azithromycin) focussed on three countries (Australia, China, Japan) (Table 1). Published data covered only 8 of the 27 WPR members (excluding states and territories potentially reporting to other WHO members). National reports were published in Australia and New Zealand.

Across both WPR and SEAR, the number of isolates for which MIC data was available for ceftriaxone increased from 8127 in 2011 to 10615 in 2016 (i.e., a 30.6% increase). The number of isolates tested for azithromycin resistance increased from 6367 in 2011 to 10803 in 2016 (i.e., a 69.7% increase). Published data was identified for 5 countries that did not provide GASP surveillance responses for ceftriaxone and 3 countries that did not provide GASP surveillance responses for azithromycin (see Table 3), although the number of isolates characterized varied. Fewer published reports contained azithromycin data as opposed to ceftriaxone data. Published reports describing ceftriaxone susceptibilities frequently did not contain MIC data. For both ceftriaxone and azithromycin, increasing participation was seen in GASP surveys. Across both regions, the number of countries and territories providing ceftriaxone MIC data increased by 183.3% from 6 in 2011 (WPR = 5, SEAR = 1) to 17 in 2016 (WPR = 12, SEAR = 5). Similarly, the number of countries and territories providing azithromycin susceptibility data increased by 183.3% from 6 in 2011 (WPR = 4, SEAR = 2) to 17 in 2016 (WPR = 12, SEAR = 5). Across both regions, published data for either ceftriaxone or azithromycin was identified for 16 of 52 searched locations, and GASP data was available for 18 of 52 locations. Overall, no ceftriaxone or azithromycin data was available for 30 locations, including 19 members of WPR or SEAR (excluding locations that potentially report to other sites).

**Table 3:** Detection of most resistant *N*. *gonorrhoeae* isolates to ceftriaxone and azithromycin in WPR and SEAR countries by evidence source (2011 to 2016).

Country	Ceftriaxone^1^	Azithromycin^2^
Australia	Alert level (GASP, National report, Publication)	Resistant (GASP, National report, Publication)
Bangladesh	Alert level (Publication)	Resistant (Publication)
Bhutan	Alert level (GASP, Publication)	Resistant (GASP)
Cambodia	No alert level (GASP)	Resistant (GASP)
China	Alert level (GASP, Publication)	Resistant (GASP, Publication)
Hong Kong	Alert level (GASP)	Resistant (GASP)
India	Alert level (Publication)	Resistant (GASP, Publication)
Indonesia	Alert level (Publication^3^)	No resistance (Publication)
Japan	Alert level (GASP, Publication)	Resistant (GASP, Publication)
Mongolia	Alert level (GASP)	Resistant (GASP)
Myanmar	Alert level (GASP, Publication^3^)	Resistant (GASP)
Nepal	No alert level (Publication)	No data
New Caledonia	No alert level (GASP)	No data
New Zealand	Alert level (GASP, National report^4^)	Resistant (GASP)
Philippines	No alert level (GASP, Publication)	No resistance (GASP)
Singapore	Alert level (GASP)	Resistant (GASP)
South Korea	Alert level (GASP, Publication)	Resistant (Publication)
Sri Lanka	No alert level (GASP, Publication)	Resistant (GASP)
Taiwan	Alert level (Publication)	No data
Thailand	Alert level (GASP, Publication^3^)	Resistant (GASP, Publication^5^)
Viet Nam	Alert level (GASP, Publication)	Resistant (GASP, Publication)

Data derived from national reports and publications interpreted based on the reported MIC, or where no MIC is provided, in the context of the stated interpretation. Sites with no data: American Samoa, Brunei, Christmas Is., Cocos Keeling Is., Cook Is., Fiji, French Polynesia, Guam, Kiribati, Lao, Malaysia, Maldives, Marshall Is., Micronesia, Nauru, Niue, Norfolk Is., North Korea, Northern Mariana Is., Palau, Papua New Guinea, Pitcairn Is., Samoa, Solomon Is., Timor-Leste, Tokelau, Tonga, Tuvalu, Vanuatu, Wallis and Futuna

1 Alert level ceftriaxone susceptibility is defined here any susceptibility result meeting or breeching the WHO criteria for decreased susceptibility (MIC ≥ 0.125 mg/L).

2 Azithromycin resistance is defined as MIC ≥ 1 mg/L.

3. Isolates reported as ‘decreased susceptibility’ although MIC breakpoint not provided.

4. Isolates reported as having reduced or decreased susceptibility with MICs ‘typically’ 0.06 mg/L and not exceeding 0.25 mg/L; rate at 0.125 to 0.25 mg/L not provided.

5. Isolates reported as ‘resistant’ although MIC breakpoint not provided.

### Rates of reported resistance

**Fig 2:**
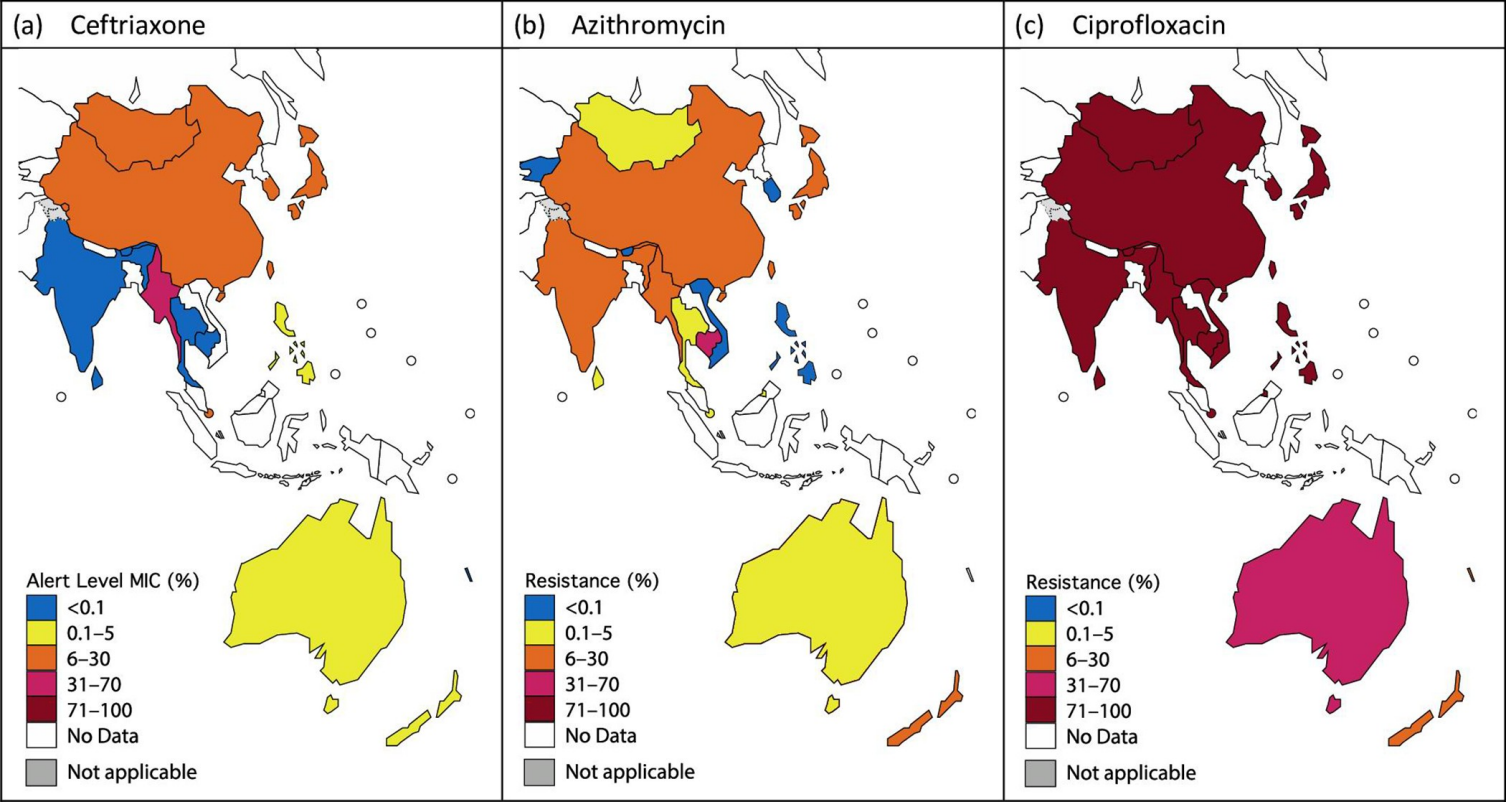
Mapping ranges for ceftriaxone, azithromycin and ciprofloxacin in the WPR and SEAR for 2016 based on surveillance data submitted to the Gonococcal Antimicrobial Surveillance Programme. (a) Ceftriaxone: Proportion of strains with MIC ≥ 0.125 mg/L; (b) Azithromycin: Proportion of strains with MIC ≥ 1 mg/L; (c) Ciprofloxacin: Proportion of strains with MIC ≥ 1 mg/L. Countries with data included irrespective of number of samples available. Countries without data excluded. Australian colouration is based on non-remote data. Ranges for Cambodia and Myanmar are based on limited (≤ 10) isolates.

The GASP survey data demonstrates considerable variation in antimicrobial mapping ranges between participant reports in both the WPR and SEAR regions for ceftriaxone, azithromycin, ciprofloxacin and penicillin (Table 2; Fig 2).

Regarding ceftriaxone, 25.0% (*n* = 17) of all survey reports from the WPR and SEAR between 2011 and 2016 demonstrated ceftriaxone alert level MICs at rates of >5%, although rates most frequently fell in the range ≤5%. Eight reports (i.e., 11.8%) demonstrated rates of alert level MICs to ceftriaxone in excess of 15%. From 2011 to 2016, the rate of occurrence of alert level MICs to ceftriaxone exceeding 5% increased from 14.3% of all survey reports in 2011 (1 from a total of 7) to 35.3% of all survey reports in 2016 (6 from a total of a total of 17).

Regarding azithromycin, the majority (76.3%) of all survey reports from 2011 to 2016 from both WPR and SEAR demonstrated rates of resistance ≤5%, with the most frequent range of resistance of isolates falling in the 0.1–5% range. However, 23.7% of all survey reports demonstrated azithromycin resistance of >5%. Resistance rates to azithromycin reaching 50% on a single country level survey report (Cambodia, 2016) were based on testing of very few isolates (*n* = 2) and are therefore difficult to interpret. Over the study period, rates of azithromycin resistance exceeding 5% increased from 14.3% of all survey reports in 2011 (1 from a total of 7) to 38.9% of survey reports in 2016 (7 from a total of 18).

With the exception of remote Australia, Fiji, and New Caledonia, ciprofloxacin resistance was uniformly high and consistently exceeded 5% across both regions, with the most common percentile band of resistance reported being 71–100%. Between 2011 and 2016, 88.7% (*n* = 94) of all survey reports from the WPR and SEAR demonstrated ciprofloxacin resistance rates exceeding 5%. Similarly, the most frequently reported mapping range in WPR and SEAR for penicillin was 31–70%, followed by 71–100%. 91.2% (*n* = 93) of all survey reports demonstrated penicillin resistance rates of >5%.

Mapping ranges could not be determined from published reports given potential publication bias. It remains unknown how frequently susceptibility testing results of isolates with lower MICs were not published versus the rate of publication of findings relating to isolates with higher MICs. However, published reports identified isolates with alert level MICs to ceftriaxone from three countries (Bangladesh, India, and Taiwan) that were not identified from GASP survey data (Table 3). Similarly, published reports identified azithromycin resistance from two countries (Bangladesh and South Korea) that were not identified from survey data.

## Discussion

The emergence of ceftriaxone and azithromycin resistance in *N*. *gonorrhoeae* represents a major global concern that carries far-reaching public health implications given no ideal alternative treatment has been identified. With increasing ceftriaxone MICs and reports of ceftriaxone resistant strains in the Asia Pacific, and subsequently in Europe and North America, azithromycin was added to ceftriaxone in dual therapy as a method of limiting the selection of ceftriaxone resistant mutants [1, 2]. Historically, the WHO has utilised a 5% threshold of antimicrobial resistance in *N*. *gonorrhoeae* to identify when alternative agents should be utilised [12]. This study confirms that the WHO 5% resistance threshold for change has been breached in many sites in the WPR and SEAR, with an increasing proportion of alert level MICs for ceftriaxone and resistance for azithromycin being observed. This observation likely reflects a global phenomenon.

The NG MIC Snapshot Study responds to pre-existing knowledge gaps and provides unique insights into the establishment of AMR in *N*. *gonorrhoeae* and its surveillance. First, via GASP survey reports, it demonstrates that between 2011 and 2016 gonococcal MICs for ceftriaxone have increased above alert levels (i.e., MIC ≥ 0.125 mg/L) with the percentage of countries, territories or other survey areas reporting rates in excess of the WHO 5% threshold rising from 14.3% to 35.3%. A greater increase has been observed regarding azithromycin resistance (i.e., MIC ≥ 1 mg/L), with the percentage of sites reporting greater than 5% resistance increasing from 14.3% to 38.9% over the same period. Detection of increasing MICs to ceftriaxone particularly in resource constrained regions was supported by the WHO CC, Sydney’s provision of MIC gradient strips and technical support to laboratories participating in the GASP survey. Over the study period, there was a 183% increase in the number of countries providing surveillance data for both ceftriaxone and azithromycin, and a 30.6% increase in ceftriaxone MIC testing across WPR and SEAR facilitated by the WHO CC, Sydney’s NG MIC Snapshot Study. MIC determination has historically been hampered by (1) the resource challenges associated with other methods of testing (e.g., microbroth dilution) particularly in areas where surveillance strengthening is most required, (2) the inability of less expensive disc-based methods to detect MIC changes, and (3) the lack of quality assessment. Second, the study uniquely integrates published literature via both structured databases and through a grey literature search to identify additional reports of emerging resistance that may not have been captured by the GASP survey. The literature search revealed additional isolates meeting alert level ceftriaxone MICs from Bangladesh, India and Taiwan, and azithromycin resistance from Bangladesh, and South Korea. Third, the combination of literature review and survey results confirm that surveillance of ceftriaxone and azithromycin remains weak in many parts of the WPR and SEAR despite this dual therapeutic combination being increasingly utilised. Additionally, the quality of published data is highly variable, with report types ranging from case reports and case series, to experimental studies, surveys and surveillance reports, and conference proceedings, and complicated by lag to publication, publication biases, difficulties in determining the precise collection date and site in various reports, variation in testing methodologies, and difficulties in assessing the quality checks performed in each report.

Various limitations should be considered when interpreting the results of this study. First, despite a comprehensive review of multiple databases, published reports may have been missed if they were not indexed by the interrogated databases, or if they were not identified in the grey literature search. Second, it is possible that susceptibility data for individual isolates was reported across multiple articles. Efforts were made to exclude articles when it was apparent that susceptibility results were republished from elsewhere. Third, the review does not account for susceptibility data published from before 2011, and does not capture data from studies published after 2016 (e.g., regarding Lao PDR, see [106]). Additionally, the review does not capture data published between 2011 and 2016 where the date and/or country of isolate collection is not explicitly indicated (e.g., [107–113]). Fourth, susceptibility results are interpreted here as initially reported; methodological variation, selection and publication bias, and undetected quality related issues in data could affect out findings. These issues extend from the regional (and global) divergence in the availability of quality AMR data based on standardised methods of assessment and reporting. Fifth, a subset of included publications describe isolate series that span beyond the study period (e.g., sample collection beginning prior to 2011 and continuing into the study period). It is possible that resistant isolates described in such reports were collected outside of the study period. Finally, this report does not include molecular studies of resistance; while this was done to ensure the comparability of data, we envisage molecular studies will play a more prominent role in the surveillance of antimicrobial resistance in *N*. *gonorrhoeae* in the future. These limitations present challenges for determining specific rates of antimicrobial resistance in the Asia Pacific.

The WHO names *N*. *gonorrhoeae* on its list of high priority pathogens given the emergence of resistance to 3^rd^ generation cephalosporin’s and fluoroquinolones [114]. Globally, there is a need for countries to engage in quality assured antimicrobial surveillance efforts for organisms such as *N*. *gonorrhoeae* that address susceptibility rates for antimicrobial agents which form the basis for national guidelines. The NG MIC Snapshot Study provides significant additional important information for the GASP, and aligns with priorities identified in the World Health Assembly’s resolution WHA68.7. It complements initiatives by the WHO including the Global Antimicrobial Resistance Surveillance System (GLASS) and the activities of the WHO AMR Surveillance and Quality Assessment Collaborating Centres Network, which is providing assistance to build capacity and develop and implement AMR surveillance, particularly in low income countries [115]. The use of gradient diffusion MIC data presented here linked with support and technical assistance to low and middle income countries by the WHO CC, Sydney has provided critical insights which enhance the situation analysis of AMR emergence in *N*. *gonorrhoeae*. For the future, we recommend that additional measures which support the GASP be utilised, including gradient strip diffusion, training, sustainability, quality practice measures and further use of molecular technologies to improve regional and global understanding.

## Supporting information

S1 Table Survey characteristics of *Neisseria gonorrhoeae* testing: 2011 to 2016. Key. WPR: Western Pacific Region; SEAR: South-East Asian Region.
(DOCX)

S1 File Gonococcal antimicrobial surveillance programme mapping ranges: 2011 to 2016. (XLSX)
