# Supplementary material for: Systematic review and survey of Neisseria gonorrhoeae ceftriaxone and azithromycin susceptibility data in the Asia Pacific, 2011 to 2016
Source: PLoS One. 2019 Apr 3;14(4):e0213312. doi: 10.1371/journal.pone.0213312 (PMC6447224; doi:10.1371/journal.pone.0213312)
Supplement: S1 Table — Key. WPR: Western Pacific Region; SEAR: South-East Asian Region. (DOCX) [file pone.0213312.s001.docx]

**S1 Table. Survey characteristics of *Neisseria gonorrhoeae* testing: 2011 to 2016.** Key. WPR: Western Pacific Region; SEAR: South-East Asian Region

|  | **Ceftriaxone** | | **Azithromycin** | |
| --- | --- | --- | --- | --- |
|  | **WPR** | **SEAR** | **WPR** | **SEAR** |
| **Minimum annual total tested** | 6880 | 546 | 5630 | 737 |
| **Maximum annual total tested** | 9763 | 970 | 9950 | 1144 |
| **Combined total for survey period** | 47654 | 4571 | 44917 | 5350 |
